# Increasing dietary fibre intake in healthy adults using personalised dietary advice compared with general advice: a single-blind randomised controlled trial

**DOI:** 10.1017/S1368980020002980

**Published:** 2021-04

**Authors:** Iris Rijnaarts, Nicole M de Roos, Taojun Wang, Erwin G Zoetendal, Jan Top, Marielle Timmer, Emily P Bouwman, Koen Hogenelst, Ben Witteman, Nicole de Wit

**Affiliations:** 1 Division of Human Nutrition & Health, Wageningen University & Research, Stippeneng 4, 6708 WE Wageningen, the Netherlands; 2 Laboratory of Microbiology, Wageningen University & Research, Wageningen, the Netherlands; 3 Wageningen Food and Biobased Research, Wageningen University & Research, Wageningen, the Netherlands; 4 Wageningen Economic Research, Wageningen University & Research, Wageningen, the Netherlands; 5 Department of Training and Performance Innovations, The Netherlands Organization for Applied Scientific Research (TNO), Soesterberg, the Netherlands; 6 Department of Gastroenterology and Hepatology, Gelderse Vallei Hospital, Ede, the Netherlands

**Keywords:** Dietary fibre, Tailored, Personalised, Evaluation, Advice

## Abstract

**Objective:**

A high-fibre diet is associated with a lower risk for diseases. However, few adults meet the dietary fibre recommendation. Therefore, the effects and acceptance of an algorithm-generated personalised dietary advice (PDA) compared with general advice (GA) on fibre intake were investigated.

**Design:**

A 6-week, single-blind randomised controlled trial with a 3-month follow-up.

**Setting:**

PDA was based on habitual intake and provided fibre-rich alternatives using a website; GA contained brochures. Dietary intake was assessed at baseline, week 1, week 6 and 3-month follow-up. Both groups evaluated their advice at week 6. All participants had access to PDA from week 7 until 3-month follow-up.

**Participants:**

Two groups of healthy adults: PDA (*n* 34) and GA (*n* 47). For 3-month follow-up analysis, participants were re-divided into visitors (*n* 52) and non-visitors (*n* 26) of the PDA.

**Results:**

At week 6, energy intake remained stable in both groups, but fibre intake per 1000 kcal increased non-significantly in both groups (PDA = Δ0·5 ± 2·8; GA = Δ0·8 ± 3·1, *P* = 0·128). Importantly, a significantly higher percentage of PDA participants adhered to the recommendation compared with week 1 (PDA = 21 % increase; GA = 4 % increase, *P* ≤ 0·001). PDA participants evaluated the advice significantly better compared with GA participants. At 3-month follow-up, fibre intake increased compared with baseline (visitors = Δ2·2 ± 2·6, *P* < 0·001; non-visitors = Δ1·5 ± 1·9, *P* = 0·001), but was insignificantly different between groups. Visitors had a decrease and non-visitors had an increase in energy intake (visitors =Δ − 132 ± 525; non-visitors = Δ109 ± 507, *P* = 0·055).

**Conclusions:**

The algorithm-generated PDA was well accepted and stimulated adherence to the recommendations more than GA, indicating to be a suitable and cost-efficient method for improving dietary fibre intake in healthy adults.

Dietary fibres play a key role in the prevention of diseases. A diet high in fibre is associated with a reduced risk for developing obesity, stroke, hypertension, diabetes and colorectal cancer^(1–5)^. Dietary fibres have been shown to delay gastric emptying time, which reduces the postprandial glucose peak and thereby prevents the development of insulin resistance: one of the causes of many chronic diseases^(6,7)^. Fibres can also increase stool weight and stool frequency and improve stool consistency, thereby supporting a healthy stool pattern^(8–13)^. They are fermented by bacteria in the colon which produces SCFA such as butyrate. Butyrate is the preferred energy source for colonocytes and known for its anti-inflammatory properties and positive effects on gut health^(11,14–16)^.

Regardless of its widely known health benefits, current fibre intakes are below the recommendations. The Health Council of the Netherlands recommends a fibre intake of 3·4 g/MJ or 14 g/1000 kcal, that is, 30 g for women and 40 g for men per d^(17)^. In the Netherlands, median fibre intake is around 18 g for women and 22 g for men^(18,19)^. Wholegrain, cereals and cereal products, vegetables, fruits, nuts and potatoes are the main food sources of fibre in the Dutch population^(19)^.

Short-term intervention studies are often successful in increasing fibre intake, but it remains difficult to sustainably increase fibre intake in large healthy populations^(20)^. Moreover, successful interventions focused often on one high-fibre group such as fruit and vegetables^(21–24)^, but did not reach the fibre recommendation, suggesting that interventions targeting single high-fibre food sources are not sufficient. Moreover, studies that use a specific – more fibre-rich – diet as intervention such as the Mediterranean diet, require major changes for a population with another dietary culture, possibly making this too complex for long-term adherence^(25,26)^.

A personal approach based on individual needs, preferences and habitual diet may be a successful strategy towards a long-term improvement of the diet. Recently, a study among Dutch seniors found beneficial effects of personalised dietary advice (PDA) compared with general advice (GA) on body fat, waist and hip circumference^(27)^. Bianchi and colleagues (2020) found that computer-based tailored dietary counselling significantly improved diet quality in eighty French pregnant women, compared with GA^(28)^. A large European trial, named Food4Me, found that PDA significantly improved healthy index scores and reduced red meat, salt and saturated fat intake compared with GA. However, PDA did not significantly improve dietary fibre, fruit, vegetables or wholegrain intake compared with GA^(29)^. Possibly, this was because dietary fibre was not the sole aim of this intervention. As far as we know, the effects of a personalised high-fibre diet were only investigated in North American children with refractory functional constipation. In that study, children received either written general dietary advice or personalised diet management by a registered dietitian. Those receiving personalised diet management showed better compliance for increasing fibre intake, water consumption, as well as energy and macronutrient intake^(30)^. This suggests that PDA is more effective than GA in improving dietary intake; however, whether this also applies for dietary fibre intake in healthy adults is unknown.

Whereas PDA interventions may be more effective than GA, they often include supervision from a nutrition counsellor, as discussed by Karagiozoglou and colleagues^(30)^. This can be time-consuming and costly, thereby limiting the potential for large-scale application. Digital interventions may form an alternative strategy, which has shown to be effective for behaviour change regarding diet^(31,32)^. In addition, behavioural change techniques may be incorporated, for example, by recommending high-fibre substitutes for habitually consumed low-fibre products or adding high-fibre products to a meal^(33)^. Research has shown that substitutions within dietary subgroups can improve nutrient adequacy^(34)^. If participants can self-select these high-fibre substitutes or add-ons, this may increase compliance to dietary advice.

To test this approach, algorithms based on dietary guidelines were developed and incorporated in a website that automatically generates PDA using input from participants on food intake and personal characteristics. We assessed whether this PDA website has an additional value besides GA in increasing fibre intake in a healthy adult Dutch population. Moreover, we evaluated how users perceived the PDA.

## Methods

**Fig. 1:**
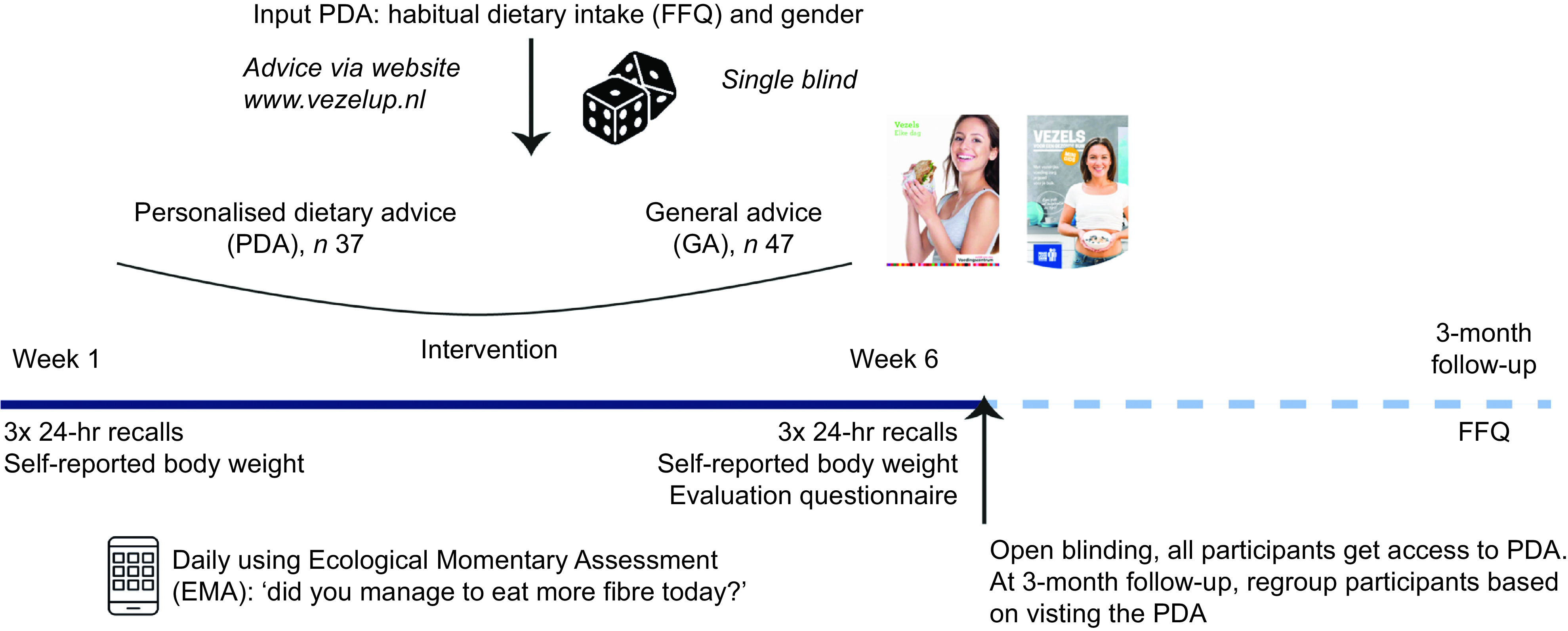
Overview of the study design. Questionnaires are performed online or via mobile application. General advice consisted of two flyers of the Netherlands Nutrition Center and the Dutch Digestive Foundation, and a website containing general information. The intervention group also received this information and their personal advice

This was a 4·5-month single-blind parallel randomised controlled trial, which included a 6-week intervention period and a 3-month follow-up. The study was performed between March and September 2019. For full overview of the study, see the CONSORT checklist. To ensure blinding, participants were not informed about advices tested in the trial and were asked not to discuss their advice with other participants. Stratified for age, gender, BMI and fibre intake before the study, participants were randomly allocated to either the GA or PDA group (ratio 1:1) by the research team. The GA consisted of two flyers: one of the Netherlands Nutrition Center and one of the Dutch Digestive Disease Foundation, and general information provided on the study website (www.vezelup.nl) about dietary fibres. The PDA group also received the GA, but had additional information on the website to compose their PDA (see below). Blinding was opened after the 6-week intervention, after which both GA and PDA participants had access to their PDA until the 3-month follow-up, to assess whether the PDA website is feasible to use without support of research staff. Figure 1 shows the study design.

The PDA was generated by modelling digitised personal and food data, which was implemented in a website (www.vezelup.nl). The advice was personalised based on dietary intake (assessed before study start using a meal-based FFQ) and gender (male/female). Generated by the algorithm and shown on the website, participants could choose high-fibre alternatives for their habitually used low-fibre products during each mealtime (breakfast, lunch, dinner and in between each meal). Prior to programming, the alternative product list was compiled by study researchers after consulting with dietitians and included general high-fibre products without using brand names (e.g. whole wheat crackers). Besides replacing low-fibre foods, participants could include an extra portion of high-fibre products such as fruit, vegetables, nuts and/or legumes at each mealtime. Participants could change their PDA freely during the 6-week intervention period, by choosing different high-fibre products or including an extra portion of high-fibre products at different mealtimes. The website displayed the participant’s current fibre, vegetables, fruits, nuts and legumes intake (based on the FFQ), their intake after choosing their PDA and a comparison with the recommendations, to serve as feedback. After choosing their PDA, participants were guided to make a so-called ‘implementation intention’. This must help attain their goals by formulating when, where and how the goal will be reached^(35)^. For the PDA group, user login data were logged to evaluate the use of the website and compliance.

### Study participants

Study participants were recruited using the Wageningen University & Research subject database. Participants were eligible if 18 years or older; apparently healthy; had a relatively low fibre intake (females <26 g, males <33 g, which is ≥15 % below the recommendation, assessed using a screening questionnaire and FFQ); were living in the Wageningen area (within 50 km radius) and were in the possession of a mobile phone compatible with required applications. Participants were excluded when they had a digestive tract disorder (chronic constipation or diarrhoea, Crohn’s disease, Ulcerative Colitis, Irritable Bowel Syndrome, Coeliac disease); presence of diabetes mellitus; were currently following a strict diet and unwilling or unable to change; using laxatives, diuretics, antidepressants, codeine, antibiotics or fibre supplements; and for female participants when currently pregnant or breast-feeding. Participants filled in the questionnaires online at home. We aimed to include at least thirty participants per group, have a power of 80 %, take a 10 % dropout rate into account and have the ability to detect a difference of 5 g/d in fibre intake^(36)^.

### Dietary assessment

Dietary fibre intake was the primary outcome of this study. During week 1 and week 6 of the intervention, dietary intake was assessed with three web-based 24-h recalls using the validated programme Compl-eat^(37)^. To reduce bias, participants were not informed beforehand when the 24-h recalls would be performed, and to take variation into account, recalls consisted of two non-consecutive workdays and one non-consecutive weekend day. Before the start of the study and at the 3-month follow-up, habitual diet of the last month was assessed with a 247-item semi-quantitative meal-based online FFQ, which was based on and developed using a validated FFQ, that also included the last month as a reference period^(38,39)^. The same items from the validated FFQ were assessed; however, in the FFQ used for this study, the items were assessed per mealtime (breakfast, during the morning, lunch, during the afternoon, dinner and during the evening). This was done to be able to give personalised advice per mealtime. Which item was assessed for which mealtime was based on the item intake of the reference population of the National Dutch Food Composition Survey^(19)^.

### Ecological momentary assessment

Ecological momentary assessment (EMA) is a structured diary technique to assess behaviour, thoughts, feelings and context in daily life^(40,41)^. Besides its common use in behavioural and social sciences, EMA has also been used to study specific dietary aspects as well as gastro-intestinal complaints^(42,43)^. In the present study, smartphone-based EMA was used daily during the intervention period to answer a fixed set of questions. This included subjective fibre intake, which was assessed daily by asking ‘did you manage to eat more fibre today?’ on a visual analogue scale ranging from 0 ‘not at all’ to 100 ‘yes, very much’. In addition, at the start and end of the intervention period, the EMA app asked participants to report their fasting body weight in the morning. Notifications to answer subjective fibre intake were sent at 20.00 hours, although participants could personalise the timing from 18.00 to 22.00 hours. Questions could be answered up to an hour after the notification. In our study, EMA compliance was high (79·1 %) during the 6-week intervention.

### Evaluation of the personalised dietary advice

After the 6-week intervention, participants completed an evaluation questionnaire to assess appreciation and acceptance of the PDA. Participants rated several aspects and statements regarding the advice on a seven-point Likert scale, ranging from 1 ‘totally disagree’ to 7 ‘totally agree’. The evaluation included advantages and disadvantages of the advice, how positive, useful, attractive or interesting they found the advice, how much the advice helped and fitted them, whether it motivated them and whether they received enough feedback.

### Statistical analysis

Data were analysed per protocol (excluding non-compliant and dropout participants). Due to the characteristics of the non-compliant and dropout participants (i.e. elderly and lack of technological skills), this group can be seen as an inappropriate target population for this intervention and therefore were excluded in the analysis. Continuous data are presented as mean ± sd or median (interquartile range) when not normally distributed. Categorical data are presented as counts and percentages. Differences between groups were tested using an independent *t* test or Wilcoxon test when not normally distributed. Differences within groups were tested using a paired sample *t* test or paired Wilcoxon test when skewed. For assessing within- and between-person variation for fibre intake of the 24-h recalls, we calculated a CV (sd/mean × 100). Regardless of the intervention group, after the 6-week intervention, all participants had access to their PDA. GA participants who visited the website after the 6-week intervention and 3-month follow-up and PDA participants were grouped together (visitors) and were compared with GA participants who did not visit the website (non-visitors), to assess the effect of the PDA and feasibility after 3 months.

To analyse EMA data, mixed linear modelling with restricted maximum likelihood estimation using lmer was used. Participants needed to complete at least 30 of 42 (75 %) of EMA days, to be included in the analysis. Treatment effects are reported using estimated least squares means and sem. Effect sizes were estimated using Cohen’s *d*. SPSS version 25 and R version 3.5 were used for testing, and a *P*-value of <0·05 was considered significant.

## Results

### Study participants

**Fig. 2:**
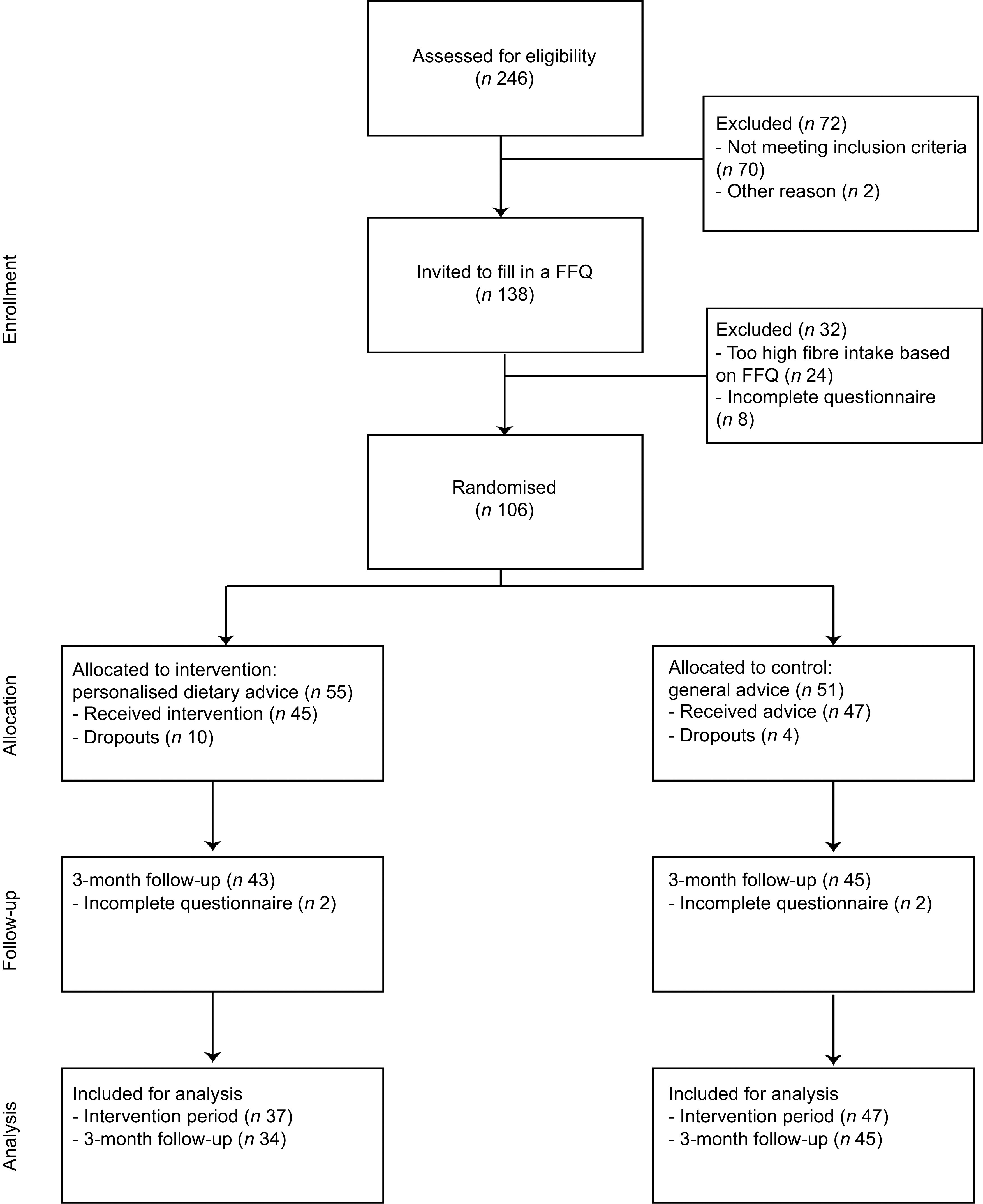
Study recruitment and flow chart

In total, 246 people were screened for the selection criteria and 106 participants were eligible, see Fig. 2 CONSORT flow chart. During the first week of the study, fourteen participants dropped out. Dropouts had an average age of 64·5 ± 15 years, a BMI of 25·8 ± 4 kg/m^2^ and 57 % was male. Moreover, of eleven participants in the PDA group, use of the website could not be confirmed by login data and therefore implementation and compliance of PDA during the intervention became uncertain. These participants were excluded from our analysis. Of these eleven participants, the median (interquartile range) age was 64 (47–68) years; they had an average BMI of 27·0 ± 5 kg/m^2^, an average fibre intake of 16·9 ± 7 g and 45 % was male. This left eighty-one subjects, of which thirty-four in the PDA group and forty-seven in the GA group, to be included for further analyses.

Table 1 shows baseline characteristics and dietary intake based on the FFQ of the included participants (*n* 81). Dietary intake, median age, percentage of males and percentage of participants with a high education level were not significantly different between the groups, but BMI was. One participant in each group met the recommendation of 14 g of fibre/1000 kcal per day, but none of the participants reached the recommendations for fibre in grams as this was an exclusion criterion.

**Table 1:** Baseline characteristics and dietary intake of the study population†

	PDA (*n* 34)		GA (*n* 47)	
	Mean	sd	Mean	sd
Age (years)				
Median	39		52	
Range	21–69		29–67	
Gender, males				
*n*	12		18	
%	35		38	
BMI (kg/m^2^)	23·7*	2·7	25·6*	4·1
Completed ≥ higher vocational education				
*n*	21		38	
%	62		81	
Energy intake (kcal)	2154	529	2015	492
Carbohydrate intake (en %)	39·5	6	38·7	5
Dietary fibre intake (g)	20·9	4	19·5	5
Dietary fibre intake (g/1000 kcal)	10·0	2·2	9·9	2·0
Water intake (ml)	2456	642	2553	625
Alcohol intake (g)				
Median	9		9	
Range	2–20		4–15	

PDA, personalised dietary advice; GA, general advice; en %, energy percentage.

* Significance between groups.

† Categorical data are presented as n and %. Dietary intake is based on a FFQ.

### Personalised dietary advice usage

The thirty-four PDA participants visited the website on average 5·6 times and made plans to change their diet 3·8 times during the 6-week intervention period. Based on their PDA, participants planned to increase their dietary fibre intake on average with 6·9 g/d. There was no significant difference in visits or number of changes made in the PDA website between males and females. From the high-fibre alternatives that could be selected on the website, 30 % of the products were chosen at least once. The five most chosen high-fibre products were fresh fruit (*n* 139), whole wheat bread (*n* 134), raw vegetables (*n* 132), nuts (*n* 130) and legumes (*n* 90).

### Body weight and dietary intake during the 6-week intervention

**Fig. 3:**
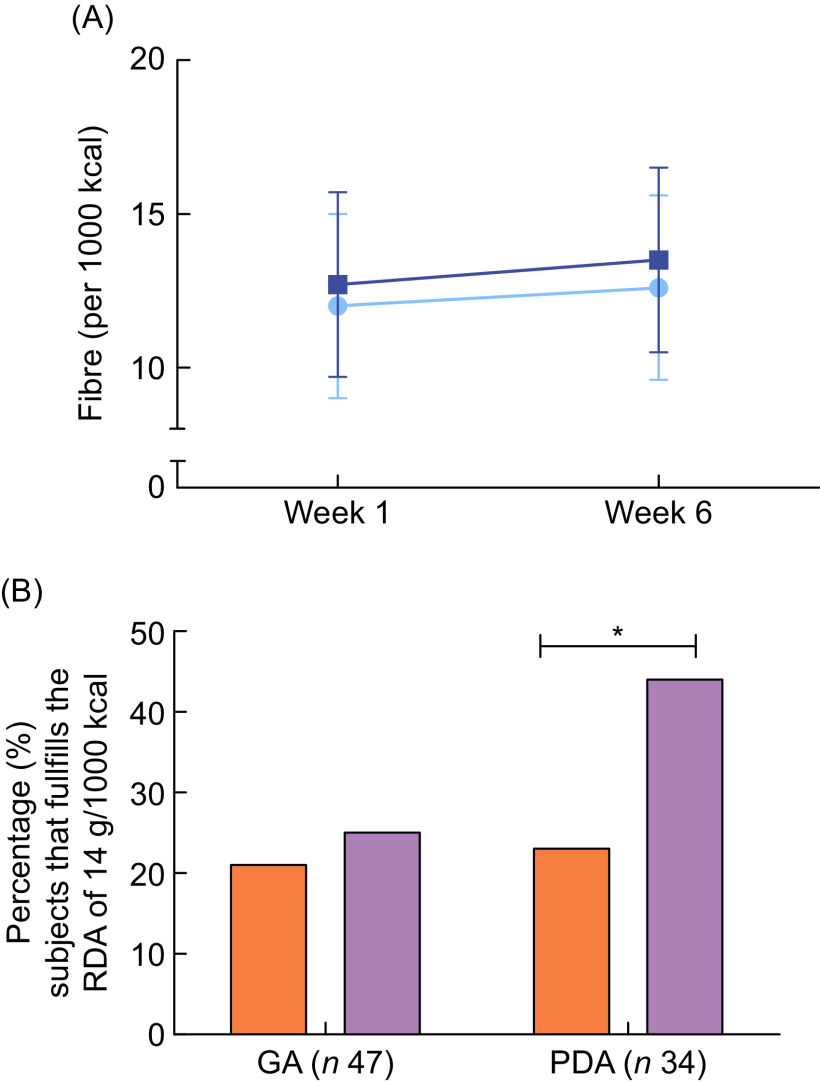
(A) Dietary fibre intake (per 1000 kcal) did not change during the 6-week intervention. Data are based on 24-h recall recalls. Error bars represent standard errors. 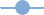, general advice (GA) (*n* 47); 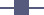, personalised dietary advice (PDA) (*n* 34). (B) Adherence to the fibre recommendation during the 6-week intervention is higher in the intervention group. Recommendation according the Dutch Health Council of 14 g of fibre/1000 kcal. 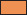, Week 1; 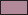, week 6. * indicates significant differences within the group

Body weight did not change substantially during the 6-week intervention period (ΔPDA = −0·25 kg, ΔGA = −0·05, *P* = 0·542), nor did energy intake (ΔPDA = −21·4 kcal (0·09 MJ), ΔGA = −21·1 kcal (0·09 MJ), *P* = 0·998), and these changes were not different between the groups. Regarding dietary fibre intake, a large within- (CV_week1_ = 28·5 %, CV_week6_ = 23·6 %) and between- (CV_week1_ = 29·6 %, CV_week6_ = 28·3 %) person variation was observed in both groups. Both groups increased intake of fibre in g/d (ΔPDA = 1·6 ± 6·4, ΔGA = 0·8 ± 6·6, *P* = 0·269), and fibre per 1000 kcal/d (ΔPDA = 0·5 ± 2·8, ΔGA = 0·8 ± 3·1, *P* = 0·128, Fig. 3A), but this was not statistically different between groups. However, importantly, a significantly higher percentage of participants in the PDA group adhered to the recommendation of 14 g/1000 kcal after 6 weeks compared with the percentage in the GA group (PDA = 21 % increase compared with baseline, GA = 4 % increase compared with baseline, *P* ≤ 0·001, Fig. 3B). To assess whether baseline fibre intake impacted effectiveness of the advice, data were stratified using median split based on the fibre intake measured by the FFQ. The change of fibre intake during the intervention period (both in g or per 1000 kcal) and number of participants adhering to the recommendations in week 6 was not different between participants with relatively low or high fibre intake (data not shown). Intended changes in dietary fibre intake based on the website did not correlate well with the change in dietary fibre intake measured by the 24-h recalls (*r* = −0·006), although both showed an increase in fibre intake during the intervention.

**Fig. 4:**
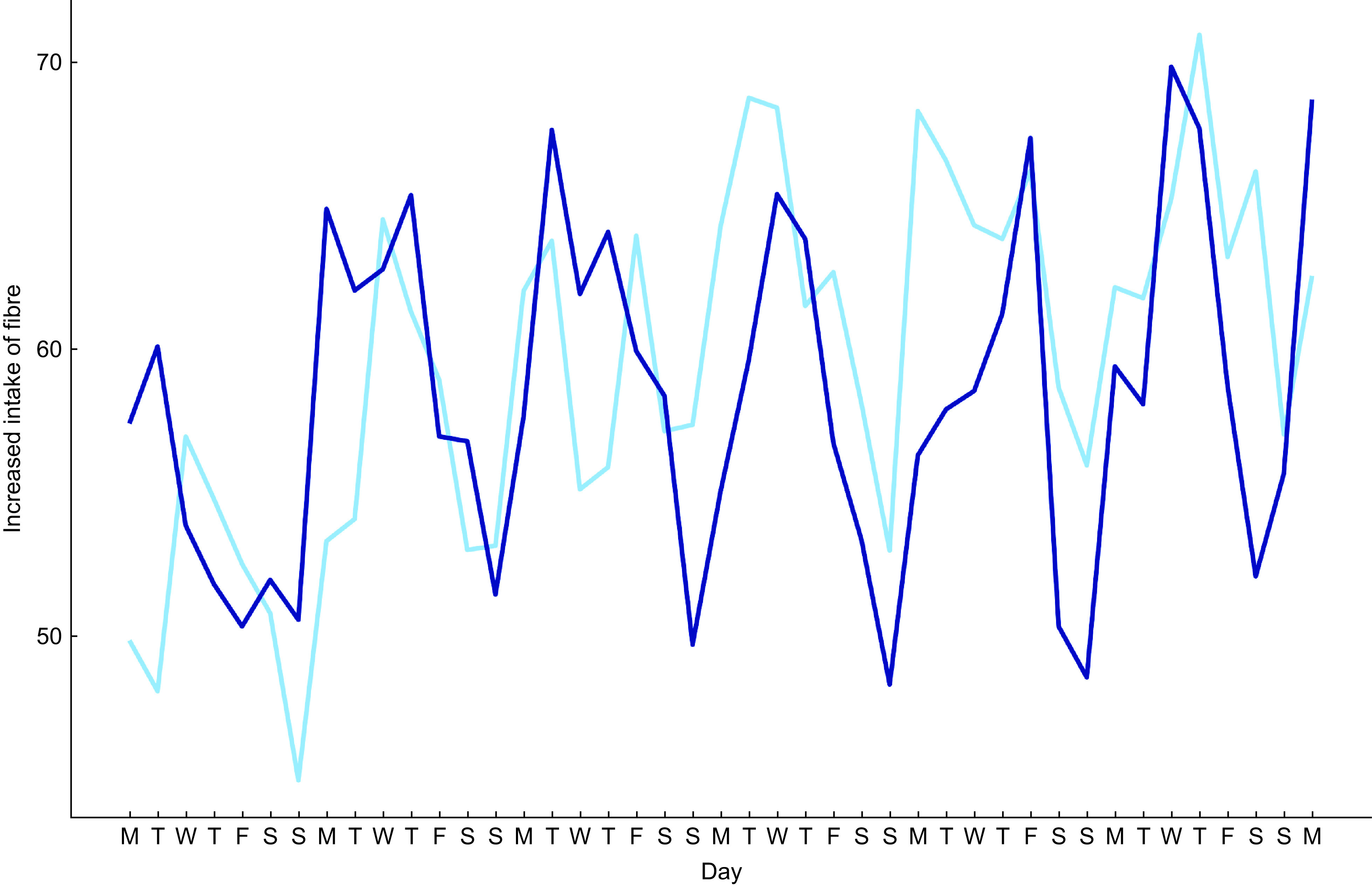
Answers to ‘did you manage to eat more fibre today’ did not differ between groups. Daily assessed using smartphone-based ecological momentary assessment (EMA). Answers were rated on a visual analogue scale rating from 0 ‘not at all’ to 100 ‘yes, very much’. 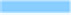, GA; 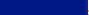, personalised dietary advice (PDA)

Daily subjective fibre intake, as assessed by EMA, did not differ significantly between groups (*P* = 0·56). Interestingly, within both groups, subjective fibre intake exhibited a consistent lower score on the weekend days (*P*’s < 0·001) (Fig. 4). The group-by-day interaction was not significant (*P* = 0·22), indicating that this weekend effect did not differ between groups. In line with EMA data, fibre intake as assessed by the 24-h recall was significantly lower on weekend days than on weekdays, both during week 1 (weekdays = 24·6 ± 7·7 g/d, Sunday =22·1 ± 9·1 g/d, *P* = 0·032) and week 6 (weekdays =25·4 ± 7·7 g/d, Sunday = 23·7 ± 8·7 g/d, *P* = 0·014). Again, this pattern did not significantly differ between groups (data not shown).

### Dietary fibre intake at 3-month follow-up

**Fig. 5:**
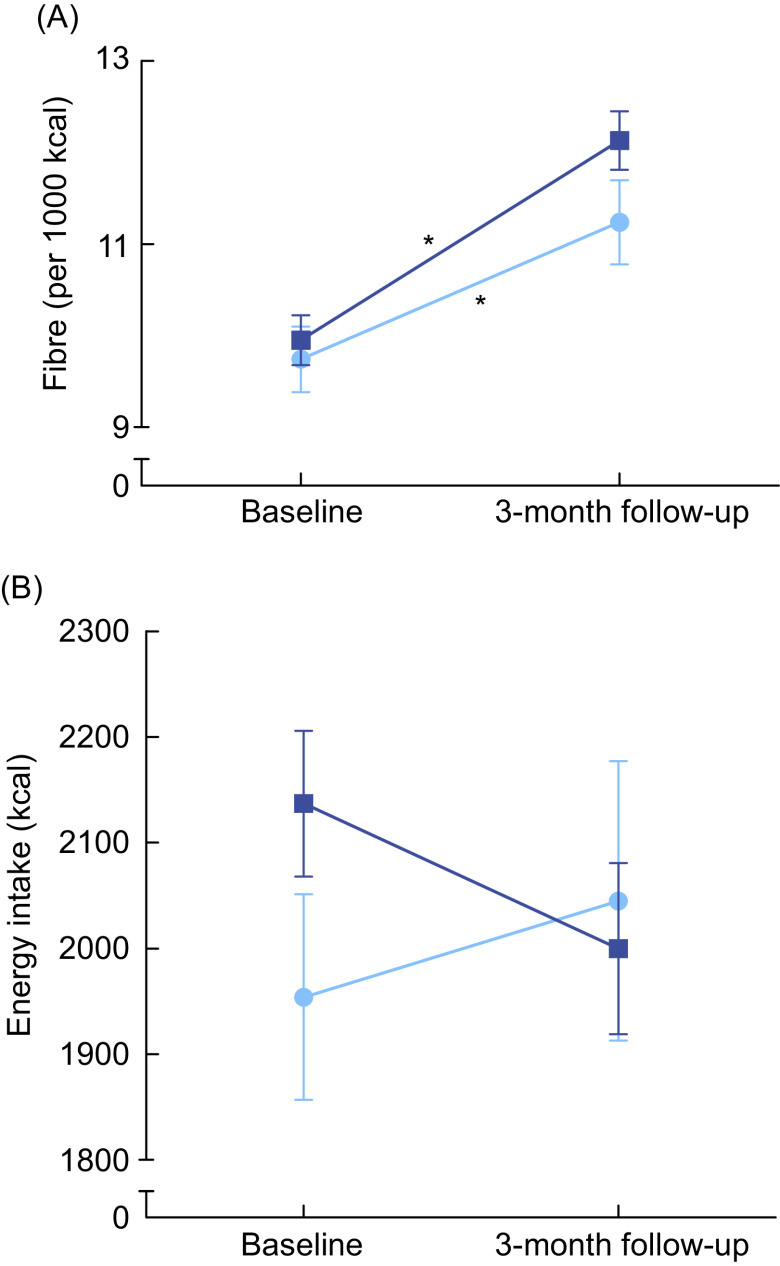
(A) Both groups increased dietary fibre intake at 3-month follow-up compared with baseline. (B) Visitors decreased their energy intake, non-visitors increased their energy intake (NS). Visitors are participants in the intervention group, and control participants who visited the personalised dietary advice (PDA) after the intervention, non-visitors are participants who never visited the PDA website. * Significant difference within the group. Error bars represent the standard errors. Data are based on the FFQ. 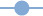, Non-visitors (*n* 26); 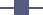, visitors (*n* 52)

After the 6-week intervention, GA participants also got access to their PDA via the website, while the PDA group maintained their access. Of the GA group, nineteen of the forty-five participants visited their PDA between the end of the intervention and the 3-month follow-up. Therefore, at the 3-month follow-up, participants were re-divided: visitors of the PDA website (*n* 52) and non-visitors (*n* 26) (*n* 4 lost to follow-up due to incomplete FFQ data, see Fig. 2). Both visitors and non-visitors significantly increased their fibre intake per 1000 kcal at 3-month follow-up compared with baseline (Δvisitors = 2·2 ± 2·6, *P* < 0·001; Δnon-visitors = 1·5 ± 1·9, *P* = 0·001, see Fig. 5A). Non-visitors had an increased daily energy intake (Δ109 ± 507 kcal (0·46 ± 2·1 MJ), *P* = 0·281) compared with baseline, whereas the visitors decreased their daily energy intake (Δ −132 ± 525 kcal (0·55 ± 2·2 MJ), *P* = 0·075, see Fig. 5B). Fibre intake was not significantly different between groups (*P* = 0·239), but the difference in energy intake was close to significance (*P* = 0·055). Visitors especially increased their fibre intake via sources of fruit (Δ0·95 g of fibre, *P* = 0·001) and legumes (Δ1·21 g of fibre, *P* < 0·000), whereas non-visitors increased their fibre intake mainly via fruit (Δ1·53 g of fibre, *P* < 0·001), vegetables (Δ1·16 g of fibre, *P* = 0·009) and nuts (Δ0·52 g of fibre, *P* = 0·001). Intake of fibre via vegetables and nuts was higher in non-visitors compared with the visitors (*P* = 0·054 and *P* = 0·052), and legumes were higher for the visitors (*P* = 0·051). For both groups, the intake of dietary fibres using wholegrain products did not significantly increase.

### Personalised dietary advice evaluation at week 6

**Fig. 6:**
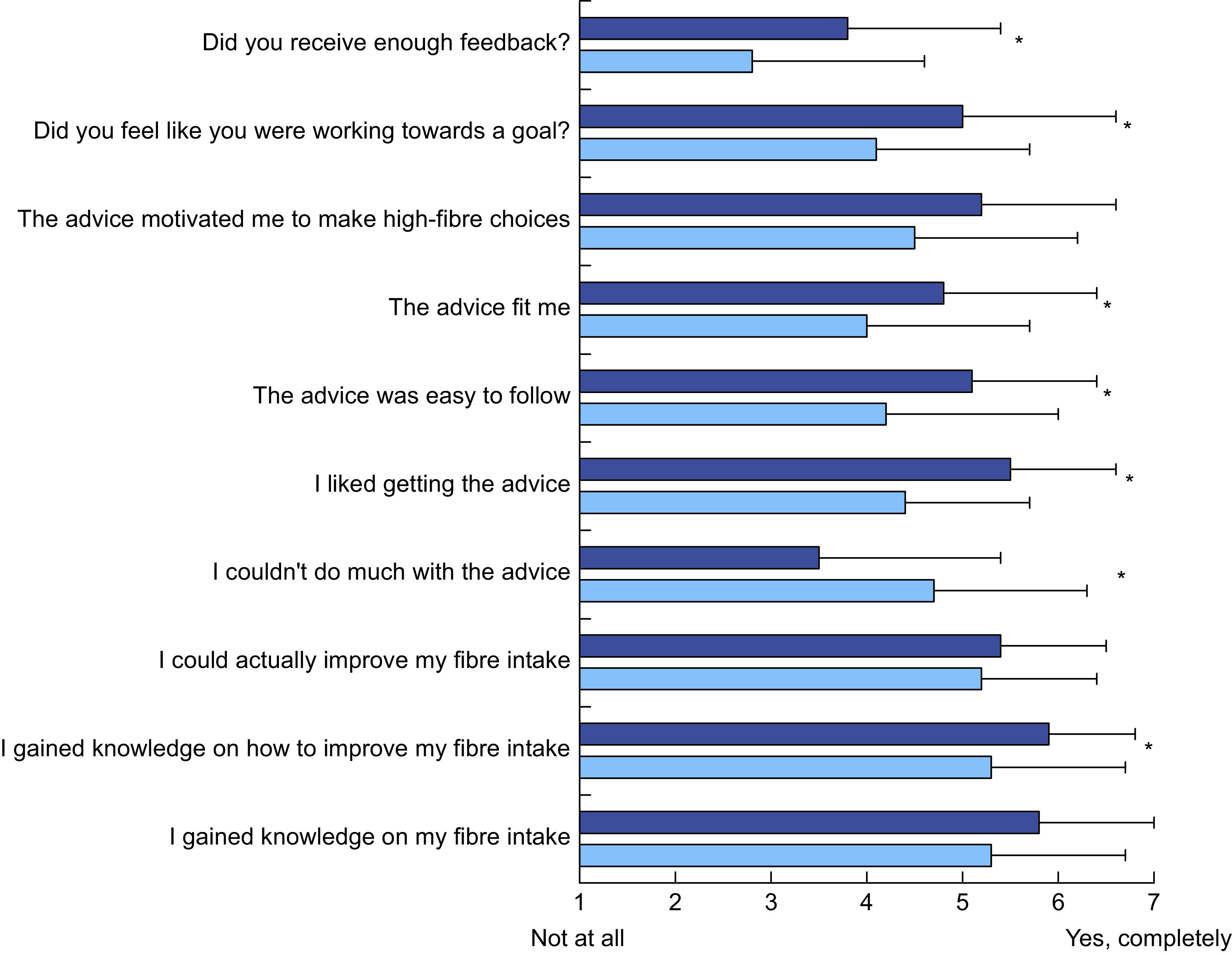
Intervention group rated the advice significantly better than the general advice (GA) group. The questionnaire was performed after the 6-week intervention. Statements were rated on a seven-point Likert scale. Error bars represent the standard deviation. 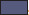, Personalised dietary advice (PDA) (*n* 34); 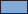, GA (*n* 47)

The PDA group rated their advice significantly better compared with the GA group regarding the following aspects of their advice: having more knowledge on how to improve their fibre intake (PDA = 5·9 ± 0·9; GA = 5·3 ± 1·4, *P* = 0·033), liking the advice (PDA = 5·5 ± 1·4; GA = 4·4 ± 1·3, *P* < 0·001), easiness of the advice to follow (PDA = 5·1 ± 1·3; GA = 4·2 ± 1·8, *P* = 0·010), motivation to make high-fibre choices (PDA = 5·2 ± 1·4; GA = 4·5 ± 1·7, *P* = 0·055), personal fit (PDA = 4·8 ± 1·6; GA = 4·0 ± 1·7, *P* = 0·032) and working towards a goal (PDA = 5·0 ± 1·6; GA = 4·1 ± 1·6, *P* = 0·021, Fig. 6). Compared with GA, PDA participants rated their advice significantly lower regarding the statement ‘I couldn’t do much with the advice’ (PDA = 3·5 ± 1·7; GA = 4·7 ± 1·6, *P* = 0·003), which indicated they perceived the advice as more positive. Although PDA participants scored significantly higher on receiving sufficient feedback, both groups had relatively low scores (PDA = 3·8 ± 1·6; GA = 2·8 ± 1·8, *P* = 0·021). The PDA group evaluated the advice significantly as more positive (PDA = 5·3 ± 1·1; GA = 4·7 ± 1·1, *P* = 0·014) and useful (PDA = 5·2 ± 1·5; GA =4·5 ± 1·4, *P* = 0·048), but there were no differences between groups in ratings of attractiveness (PDA =4·7 ± 1·2; GA = 4·3 ± 1·4, *P* = 0·187) or interestingness (PDA = 4·7 ± 1·4; GA = 4·3 ± 1·4, *P* = 0·295). There was no significant difference between the PDA and GA regarding general satisfaction of the study (PDA = 5·5 ± 0·9; GA = 5·4 ± 0·9, *P* = 0·435) or self-perceived gained knowledge about fibres (PDA = 5·8 ± 1·2; GA = 5·3 ± 1·4, *P* = 0·094).

## Discussion

In this study, we showed that a PDA generated by an algorithm and provided by a website has additional value compared with GA in increasing dietary fibre intake in healthy adults. Interestingly, the absolute amount of fibre in g/d did not increase significantly, but the percentage of people adhering to the recommendation per 1000 kcal did, indicating that people in the PDA group ate more fibre in the same amount of consumed energy than people receiving GA. Moreover, the algorithm-generated PDA was evaluated more positively than GA, indicating that website-based PDA is well accepted.

Several studies investigating PDA find similar positive results. Brinberg and colleagues (2000) performed a four-arm face-to-face high-fibre advice intervention including a group that received a tailored message, general message with intake feedback, general message with no feedback or a control group that received no message. Messages were given once at the start of the intervention, and effects were measured 6 months later. They found that participants who received a tailored message significantly increased their dietary fibre intake and dietary fibre food knowledge, but did not find an effect on food choices, compared with the other levels of intervention^(44)^. Bianchi and colleagues (2020) investigated the effects of computer-based tailored dietary counselling with a dietitian compared with general dietary counselling in eighty French pregnant women. The tailored advice was provided during counselling appointments with a dietitian and was generated using software that gave three options for improvement of the nutrient adequacy score. They found that the tailored advice was able to significantly increase the nutrient adequacy scores, while the GA did not^(28)^. The same research group has found that substituting food items within the same subgroup improved nutrient adequacy and was moderately acceptable, indicating that food substitution within food groups is a valid method to increase diet quality and still acceptable^(45)^. Moreover, in a study in eighty-six children with refractory constipation, a face-to-face personalised high-fibre, high-water intervention prescribed by a dietitian was more successful in increasing dietary fibre intake compared with general written instructions from a physician^(30)^.

The above-mentioned interventions all included face-to-face counselling, but this may not be feasible for a larger population. One of the larger internet-delivered PDA studies was the Food4Me trial, which included 1269 participants. They investigated three different levels of personalising advice, namely based on (1) individual diet, (2) individual diet, anthropometry and biomarkers and (3) as level 2 + genotype, compared with GA, aiming to improve overall diet. There was no difference between the PDA levels, but they have shown positive effects of PDA compared with GA in regard to healthy eating index scores, salt, saturated fat and red meat consumption, but not for dietary fibre intake, fruit, vegetable and whole wheat intake^(29)^. Possibly, this is due to that daily intakes of fibre-rich products such as fruit (378 g), vegetables (221 g) and wholegrains (164 g) were already high before start of the intervention, leaving little room for improvement. Moreover, dietary fibre was not the sole aim of the intervention, and fibre intake per 1000 kcal, which showed the most pronounced improvement in our study, was not reported^(29)^.

Our study is the next step in internet-delivered PDA, since it was one of the first to use a product-level model as input, making it more feasible to reach a larger population. However, previous studies as well as ours do not show effectiveness of PDA in terms of absolute fibre intake, possibly due to small differences with GA. Moreover, an accurate estimate of dietary intake remains challenging, partly due to the large within- and between-person variation.

This large within- and between-person variation was also found in our study (25–30 %) when assessing fibre intake from 24-h recalls. We only found a subtle and non-significant increase in fibre intake during the 6-week intervention. This can partly be explained by our 24-h recall timing, since we measured a few days into week 1 of the intervention. Probably participants were enthusiastic and increased their fibre intake already within those first days of the intervention, making it not a real baseline fibre intake measurement. This assumption is supported by the fact that fibre intake measured by the FFQ 2 weeks before the start of the study was lower compared with fibre intake measured by 24-h recalls during week 1 (3·9 g). Fibre intake measured by the FFQ showed that only two participants met the recommendation per 1000 kcal at the start, but 24-h recall data suggested that eighteen participants adhered to the recommendation of fibre. Although differences between the FFQ and 24-h recalls have been reported before, most often the FFQ had a higher estimate of fibre intake than 24-h recalls^(46,47)^. This indicates that the increase in fibre during the 6-week intervention, estimated by 24-h recall data, is probably underestimated in this study. However, this is likely to apply to both the PDA and GA groups. The underestimation of the change in fibre intake based on the 24-h recalls during the intervention is further supported by our 3-month follow-up FFQ, in which both non-visitors and visitors increased their fibre intake compared with baseline FFQ. This indicates that participants did substantially and significantly increase their fibre intake during the intervention period.

At the 3-month follow-up, although non-significant, visitors had a higher fibre intake and a lower energy intake compared with non-visitors. Compared from baseline to 3-month follow-up, visitors increased fibre intake by increasing fruit intake and legumes intake and non-visitors increased their fruit, vegetable and nut intake. However, by using the FFQ, we may have missed some of the true changes the PDA participants made. Many of the high-fibre alternatives generated by the PDA are not included in the FFQ, such as hummus (chickpea spread), quinoa and wholegrain options for rice and pasta. The FFQ used is based on the intake of the reference population from the National Dutch Food Composition Survey^(19)^, and these products were not frequently enough consumed by the Dutch population between 2007 and 2010 to be included in the FFQ. Therefore, fibre intake in visitors may be underestimated, and the intervention may be more effective than we measured. It is important to note that visitors and non-visitors were not randomly allocated, and it is uncertain whether visiting the website caused the differences between these groups, or whether other factors such as higher motivation in visitors resulted in this difference. Due to our study design, we could not assess this.

Based on our experience, some factors need to be considered when designing a PDA delivered via a digital tool such as a website. As shown by our dropouts and ‘non-login participants’, age and technological skills of the population seem to be important to consider beforehand. Most reported reasons for dropouts were technological difficulties and time investment needed for the study (*n* 12, 85 %). Although we provided a paper manual and instruction videos to facilitate website use, this may not be sufficient to prevent and overcome technical difficulties. However, in our study, the reported difficulty of technology may not be solely pointed to the PDA website; a previous study in Dutch seniors >60 years did not report any issues for PDA use via a website^(27)^. However, in that study, less input was required because the intake and the advice were given at the more general level of food categories. This makes the advice less ‘actionable’. Moreover, since we also used other technologies for study measurements (EMA application, FFQ and 24-h recall websites), a combination of several different technologies together might have been the reason to drop out.

Limitations of this study include the self-reported outcomes such as dietary intake and evaluation which are both sensitive to variability and social desirable answers. However, currently no valid biomarker for dietary fibre intake exists. Plasma alkylresorcinol is proposed as a biomarker for wholegrain intake, but total fibre intake as well as other grain sources such as oats, barley, maize or rice was not correlated with this biomarker^(48)^. Regarding social desirability, participants performed questionnaires online, which reduces social desirability as compared with face-to-face questionnaires^(49)^. However, due to these web-based platforms, we also encountered some technological problems (such as errors when logging-in) during the study, which may have influenced our results.

An important strength of this study is the single-blinded randomised controlled trial design, which reduces bias. Moreover, the relatively large sample size enabled us to assess effects of the intervention. In addition, the 3-month follow-up allowed us to assess whether PDA participants maintained a high-fibre intake after the intervention and thus whether PDA can provoke a sustainable long-term change in dietary fibre intake. To our knowledge, this is the first personalised high-fibre dietary advice study integrating personal and food data knowledge into an algorithm and thereby modelling advice to improve fibre intake in healthy adults, by allowing participants to choose their own high-fibre alternatives.

## Conclusion

This study showed that an algorithm-generated PDA that was delivered via a website is an accepted method to empower people to make sustainable changes in their diet. PDA helped significantly more people to adhere to the fibre recommendation than GA, especially as it increased fibre intake combined with a reduced energy intake after 3 months. Remarkably, there was significantly lower fibre intake during weekend days than on weekdays for both groups. Several aspects such as technological support and highly reproducible dietary assessment are important for effectiveness and validation of the PDA. As our study mainly included well-educated healthy adults, future studies should evaluate the effectiveness of PDA in other populations such as in participants with low-socioeconomic status, or in participants with gastro-intestinal complaints such as constipation.
